# Increased MicroRNA-146a Levels in Plasma of Patients with Newly Diagnosed Type 2 Diabetes Mellitus

**DOI:** 10.1371/journal.pone.0073272

**Published:** 2013-09-02

**Authors:** Ying Rong, Wei Bao, Zhilei Shan, Jun Liu, Xuefeng Yu, Songfan Xia, Hui Gao, Xia Wang, Ping Yao, Frank B. Hu, Liegang Liu

**Affiliations:** 1 Department of Nutrition and Food Hygiene, Hubei Key Laboratory of Food Nutrition and Safety, School of Public Health, Tongji Medical College, Huazhong University of Science and Technology, Wuhan, China; 2 Ministry of Education Key Laboratory of Environment and Health, School of Public Health, Tongji Medical College, Huazhong University of Science and Technology, Wuhan, China; 3 Division of Endocrinology, Department of Internal Medicine, Tongji Hospital, Tongji Medical College, Huazhong University of Science and Technology, Wuhan, China; 4 Medical Examination Center, Wuhan Pu’ai Hospital, Tongji Medical College, Huazhong University of Science and Technology, Wuhan, China; 5 Department of Nutrition and Epidemiology, Harvard School of Public Health, Boston, Massachusetts, United States of America; Louisiana State University Health Sciences Center, United States of America

## Abstract

**Background:**

MicroRNAs (miRNAs), a class of small non-coding RNAs, are thought to serve as crucial regulators of gene expression. Dysregulated expression of miRNAs has been described in various diseases and may contribute to related pathologic processes. Our aim was to examine circulating miRNA-146a levels in newly diagnosed type 2 diabetes mellitus (new-T2DM) patients from a Chinese Han population.

**Methodology/Principal Findings:**

Circulating miRNA-146a was extracted from plasma samples of 90 new-T2DM patients and 90 age- and sex-matched controls. Quantitative PCR assessment revealed that circulating miRNA-146a levels were significantly elevated in new-T2DM patients compared with controls. Participants in the highest tertile of circulating miRNA-146a levels showed a notably higher risk for new-T2DM (crude OR 4.333, 95% CI, 1.935 to 9.705, *P* = 0.001) than persons in the lowest tertile. Controlling for known risk factors and some biochemical indicators did not attenuate the aforementioned association. In addition, receiver operating characteristic (ROC) curves generated for miRNA-146a revealed an area under the curve (AUC) of 0.725 (95% CI, 0.651 to 0.799, *P* < 0.001). Moreover, higher circulating miRNA-146a levels were significantly associated with higher plasma heme oxygenase-1 (HO-1) concentrations (β coefficient = 0.131, *P* < 0.001) and lower HOMA-beta (β coefficient = -0.153, *P* = 0.015).

**Conclusions/Significance:**

We found that circulating miRNA-146a levels were significantly elevated in new-T2DM patients compared with healthy controls. Whether expression of circulating miRNA-146a holds predictive value for T2DM warrants further investigations.

## Introduction

Since their discovery by Lee and colleagues [1], microRNAs (miRNAs), a class of endogenous small non-coding RNAs, are becoming increasingly recognized as crucial regulators in gene expression networks [2,3]. They can function as translational repressors by binding to the 3’ untranslated regions (3’ UTRs) of target messenger RNAs (mRNAs) and promoting their degradation and/or directing posttranscriptional repression [2,4]. In the past several years, accumulating evidence suggests that miRNAs are not only key regulators in normal physiological processes, but also play an important role in the pathologic processes of cardiovascular disease, diabetes, and cancers [5–9]. Recently, extracellular miRNAs were shown to have stable levels, and circulating miRNAs have been detected in serum, plasma, salvia, urine, and other body fluids [10–12]. It has been suggested that exosomes or protein complexes protect circulating miRNAs from plasma endogenous RNases [12–14]. Interestingly, the expression profiles of miRNAs have been shown to differ considerably between patients and healthy controls [15,16]. To date, miRNAs have been investigated as biomarkers in several diseases [17,18] including type 2 diabetes mellitus (T2DM) [19].

T2DM is a global public health crisis, affecting millions of people in both developed and developing countries. By 2030, the number of diabetic patients worldwide is expected to reach 438 million, and the economic cost is projected at $490 billion per year [20]. Pathogenesis of T2DM involves enhanced oxidative stress caused by iron metabolism [21]. Heme oxygenase-1 (HO-1) is the rate-limiting enzyme that degrades heme to carbon monoxide (CO), ferrous iron and biliverdin, finishing with catalysis by biliverdin reductase [22,23]. HO-1 seems to be a novel protective factor with potent anti-inflammatory, anti-oxidant, and anti-proliferative effects [24]. Our previous research suggested that plasma HO-1 concentration was elevated in individuals with new-T2DM [25]. Among the miRNAs identified so far, miRNA-146a has been shown to be involved in the expression of HO-1 [26]. To date, there are limited data on the association between circulating miRNA-146a levels and risk of T2DM. Therefore, our aim was to examine circulating miRNA-146a levels in relation to risk of T2DM and plasma HO-1 concentrations in a Chinese population.

## Methods

### Study Population

This study included 90 new-T2DM cases and 90 age- and sex-matched controls. Cases were recruited from patients visiting the outpatient clinics of the Department of Endocrinology of Tongji Hospital (affiliated with Tongji Medical College) between March 2011 and June 2011. Controls came from an otherwise unselected population undergoing routine health examinations in the medical examination center of Wuhan Pu’ai Hospital (also affiliated with Tongji Medical College) between July 2011 and October 2011. The inclusion criteria for both cases and controls were: age ≥30 years, body mass index (BMI) <40, and no history of diagnosis of diabetes. Individuals with any other clinically systemic acute or chronic inflammatory diseases, acute respiratory infection, or cancers were excluded. All subjects were of Chinese Han ancestry. The study was performed in accordance with the Declaration of Helsinki and approved by the Medical Ethics Committee of Tongji Medical College. Written informed consent was obtained from all subjects. A standard questionnaire was used to collect information about age, sex, smoking, alcohol drinking, history of hypertension, and family history of diabetes in first-degree relatives. Standardized techniques were used for anthropometric measurements including height (m) and weight (kg). Body mass index (BMI) was calculated as [weight (kg)/height (m)^2^]. All subjects underwent a general physical exam the morning after an overnight fast, and fasting blood samples were drawn for plasma separation.

### Laboratory Measurements

Plasma levels for biochemical parameters, including fasting plasma glucose (FPG), 2-h post-glucose load (OGTT2h), fasting plasma insulin (FPI), total cholesterol (TC), triglycerides (TG), high-density lipoprotein cholesterol (HDL-C), and low-density lipoprotein cholesterol (LDL-C) were measured as previously described [27]. Beta cell function (HOMA-beta) and insulin resistance (HOMA-IR) as indicators of homeostasis model assessment were utilized to assess the status of insulin secretion and insulin action, respectively. HOMA-beta = 20* FPI (mU/ml)/[FPG (mmol/l)-3.5], HOMA-IR = FPG (mmol/l)* FPI (mU/ml)/22.5 [28]. Plasma HO-1 levels were determined using the HO-1 human ImmunoSet Kit (EKS-800, Stressgen Bioreagents; now Enzo Life Sciences, Farmingdale, NY, USA).

### Ascertainment of New-T2DM

T2DM was diagnosed by fasting glucose and/or oral glucose tolerance test (OGTT) according to the World Health Organization criteria of 1998 [29]. Participants with fasting plasma glucose ≥ 7.0 mmol/l (126 mg/dl) and/or OGTT2h ≥ 11.1 mmol/l (200mg/dl) were designated as having T2DM. Controls were subjects with fasting plasma glucose < 6.1 mmol/l (110 mg/dl) and OGTT2h < 7.8 mmol/l (140mg/dl). Self-reported T2DM status was confirmed by OGTT results. None of the participants reported taking any anti-diabetic medications.

### MiRNA Expression from Plasma Samples

Bloods were collected from participants in EDTA-coated tubes and processed for plasma isolation within 2 hours of collection. MiRNAs were isolated from 300 μl of plasma using the *mir*Vana™ PARIS™ Kit (Applied Biosystems, Foster City, CA, USA), which uses a denaturing and phenol chloroform extraction approach. Briefly, 300 μl of plasma was added to an equal amount of denaturing solution and incubated on ice for 5 minutes. Afterwards, 600 μl of acid-phenol chloroform solution was added to the samples to inhibit the activity of RNAases and accelerate the separation of layers. The aqueous phase was passed through glass-fiber filters to immobilize the RNA, which was washed a few times and eluted with a low ionic-strength solution, yielding a final volume of 80 μl. Then the TaqMan MicroRNA Reverse Transcription Kit (Applied Biosystems, Foster City, CA, USA) was used for reverse transcription. Finally, miRNAs were quantified by real-time PCR using TaqMan miRNA assays (Applied Biosystems, Foster City, CA, USA), according to the manufacturer’s instructions. Currently, there is no consensus on the selection of endogenous control for investigating plasma miRNA levels using real-time PCR. Some researchers have suggested that miRNA-16, which was previously validated in cancer patients, seems to be a reliable internal control for circulating miRNAs [12]. Therefore, our data were standardized to plasma miRNA-16 levels. Meanwhile, triplicate samples were used throughout. Relative expression of miRNA was calculated using the 2^-ΔΔCt^ method [30]. The Ct values from real-time PCR assays greater than 35 were treated as not expressed.

### Statistical Analysis

Comparisons between cases and controls were computed by Chi-square (categorical variables), student t test (continuous variables, normal distribution), or Mann-Whitney U test (continuous variables, skewed distribution). Plasma HO-1 concentrations, circulating miRNA-146a levels, and HOMA-beta were natural log–transformed prior to general linear regression analysis. Multivariate logistic regression analysis was used to assess the association of new-T2DM with miRNA-146a levels. For calculation of odds ratios (ORs) and 95% confidence intervals (CI) for new-T2DM, circulating miRNA-146a levels and plasma HO-1 concentrations were categorized into tertiles based on the distribution in the control group. ORs and 95% CIs were adjusted for known risk factors of T2DM, including age, sex, BMI, smoking, alcohol drinking, history of hypertension, family history of diabetes, and some biochemical indicators. Area under the curve (AUC) using receiver operating characteristic (ROC) analysis was calculated for miNRA-146a to assess predictive value. A 2-tailed *P* value < 0.05 was considered significant. The differences in areas under ROC curves for logistic regression models were conducted with STATA version 11 (Stata-Corp, College Station, Texas), and all other statistical analyses were performed using SPSS for Windows software version 12.0 (SPSS Inc, Chicago, IL, USA).

## Results

This study consisted of 90 new-T2DM cases and 90 age- and sex-matched controls. Demographic and clinical characteristics of the study subjects are shown in Table 1. BMI was significantly higher in new-T2DM patients than in controls. More controls than new-T2DM patients were alcohol drinkers. However, the percentage of persons with hypertension and family history of diabetes differed significantly between cases and controls. In addition, cases and controls had statistically different laboratory results for TG (*P* = 0.040), FPG, FPI, HOMA-beta, HOMA-IR, and plasma HO-1 concentrations (*P* < 0.001).

**Table 1:** Characteristics of the participants*.

Characteristics	New-T2DM patients	Controls	*P* value
No. of subjects	90	90	
Age, years	48.50 (42.00-56.00)	48.00 (41.75-55.00)	0.416
Male, n (%)	47 (52.22)	47 (52.22)	1.000
BMI, kg/m^2^	24.58 (3.66)	23.38 (2.95)	0.016
Smoking, n (%)	26 (28.89)	35 (38.89)	0.156
Alcohol drinking, n (%)	14 (15.56)	26 (28.89)	0.031
FH of Diabetes, n (%)	24 (26.67)	11 (12.22)	0.014
Hypertension, n (%)	32 (35.56)	17 (18.89)	0.012
TC (mmol/l)	4.98 (1.06)	4.83 (1.01)	0.342
TG (mmol/l)	1.47 (1.10-2.14)	1.35 (1.01-1.79)	0.040
HDL-C (mmol/l)	1.06 (0.93-1.24)	1.13 (0.88-1.38)	0.375
LDL-C (mmol/l)	3.19 (0.93)	3.16 (0.97)	0.828
FPG (mmol/l)	7.41 (6.76-12.37)	5.24 (5.01-5.53)	<0.001
FPI (μU/ml)	9.20 (5.42-14.92)	6.18 (4.74-8.76)	<0.001
HOMA-beta	37.82 (19.98-71.70)	74.72 (47.28-113.94)	<0.001
HOMA-IR	3.47 (2.04-5.50)	1.52 (1.10-2.08)	<0.001
HO-1 (ng/ml)	2.02 (1.68-2.47)	1.63 (1.13-1.98)	<0.001

* Data are presented as number (percentage) for categorical data, mean (standard deviation) for parametrically distributed data, or median (interquartile range) for nonparametrically distributed data.

Abbreviations: BMI, body mass index; FH, family history; FPG, fasting plasma glucose; FPI, fasting plasma insulin; HDL-C, high-density lipoprotein-cholesterol; HO-1, heme oxygenase-1; HOMA-beta, homeostasis model assessment of beta cell function; HOMA-IR, homeostasis model assessment of insulin resistance; LDL-C, low-density lipoprotein-cholesterol; New-T2DM, newly diagnosed type 2 diabetes mellitus; TC, total cholesterol; TG, triglycerides.

**Figure 1:**
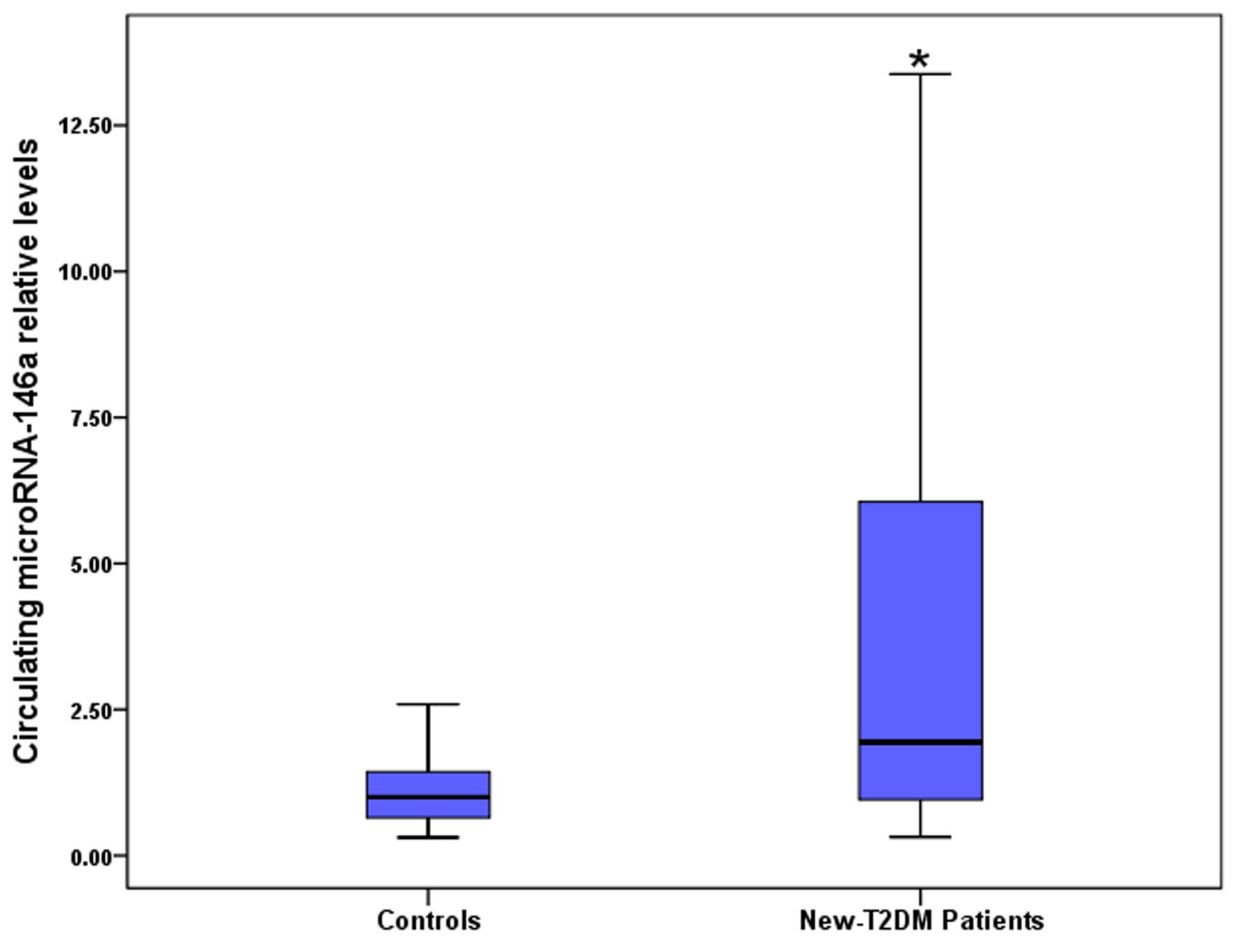
Circulating miRNA-146a levels in controls and new-T2DM patients. Data are shown as median (interquartile range). ^*^ *P* < 0.05 compared to controls. Abbreviations: new-T2DM, newly diagnosed type 2 diabetes mellitus.

Using miRNA-16 as a normalization control, we found a more robustly elevated expression of circulating miRNA-146a in participants with new-T2DM than among controls (*P* < 0.001) (Figure 1). Subsequently, we assessed the ORs for new-T2DM associated with circulating miRNA-146a levels, which were categorized into tertiles according to their distribution among the controls (Table 2). Increased ORs were observed for new-T2DM in relation to higher level of circulating miRNA-146a levels. Participants in the highest tertile of circulating miRNA-146a levels were at significantly elevated risk for new-T2DM (crude OR 4.333, 95% CI, 1.935 to 9.705, *P* < 0.001) compared with persons in the lowest tertile. The result was not altered after adjusting for age, sex, and BMI or further for smoking, alcohol drinking, disease history, and some biochemical indicators. Additional adjustment for plasma HO-1 concentrations slightly attenuated the above relationship (adjusted OR 3.832, 95% CI, 1.505 to 9.759, *P* = 0.010).

**Table 2:** Odds ratios (95% CI) for new-T2DM, by tertiles of plasma miRNA-146a levels.

Variable	Tertiles of plasma miRNA-146a levels			*P* - trend
	1 (Lowest)	2	3 (Highest)	
**MiRNA-146a levels**	<0.740	0.740-1.339	≥1.339	
New-T2DM patients/Controls, n/n	12/30	26/30	52/30	
Crude OR (95% CI)	1	2.167 (0.925-5.074)	4.333 (1.935-9.705)	0.001
Adjusted OR (95% CI), Model 1*	1	2.413 (0.997-5.842)	4.938 (2.129-11.456)	<0.001
Adjusted OR (95% CI), Model 2*	1	2.312 (0.918-5.825)	4.917 (2.044-11.828)	0.001
Adjusted OR (95% CI), Model 3*	1	1.978 (0.760-5.151)	4.815 (1.968-11.780)	0.001
Adjusted OR (95% CI), Model 4*	1	1.757 (0.646-4.781)	3.832 (1.505-9.759)	0.010

* Model 1, adjusted for age, sex and BMI; Model 2, adjusted for Model 1, smoking, alcohol drinking, family history of diabetes and hypertension; Model 3, adjusted for Model 2, TC, TG, HDL-C and LDL-C; Model 4, adjusted for Model 3 and HO-1 concentration.

Abbreviations: BMI, body mass index; CI, confidence interval; HDL-C, high-density lipoprotein-cholesterol; HO-1, heme oxygenase-1; LDL-C, low-density lipoprotein-cholesterol; New-T2DM, newly diagnosed type 2 diabetes mellitus; OR, odds ratio; TC, total cholesterol; TG, triglycerides.

**Figure 2:**
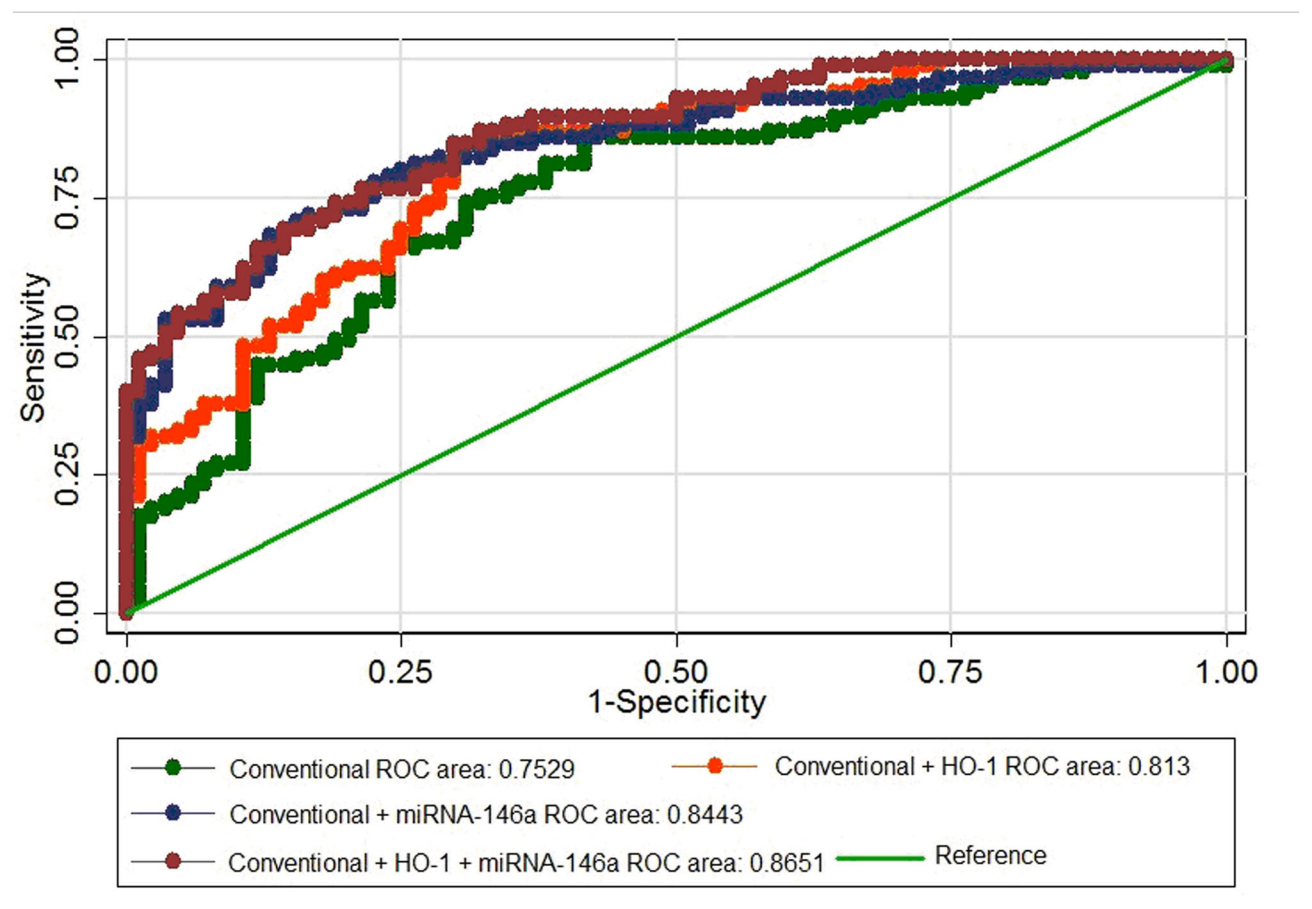
ROC curves and corresponding AUCs for new-T2DM. The AUC in an established conventional model was 0.753 (95% CI, 0.679 to 0.827), and that in a model with plasma HO-1 concentration plus the established conventional model was 0.813 (95% CI, 0.750 to 0.876). After circulating miRNA-146a levels were introduced, the AUC was 0.844 (95% CI, 0.785 to 0.903). In addition, the AUC of all parameters included was 0.865 (95% CI, 0.813 to 0.917); *P* = 0.0009 for the differences of the above AUCs. The established conventional model here consists of age, sex, BMI, smoking, alcohol drinking, hypertension, and family history of diabetes, TC, TG, HDL-C and LDL-C. Abbreviations: AUC, area under the curve; BMI, body mass index; CI, confidence interval; HDL-C, high-density lipoprotein-cholesterol; HO-1, heme oxygenase-1; LDL-C, low-density lipoprotein-cholesterol; new-T2DM, newly diagnosed type 2 diabetes mellitus; ROC, receiver operating characteristic; TC, total cholesterol; TG, triglycerides.

In the ROC analysis, miRNA-146a distinguished new-T2DM patients from controls with an AUC of 0.725 (95% CI, 0.651 to 0.799, *P* < 0.001). We then performed the comparisons among models with or without plasma HO-1 concentrations and circulating miRNA-146a levels (Figure 2). The AUC for the conventional model, comprising age, sex, BMI, smoking, alcohol drinking, history of hypertension, family history of diabetes, TC, TG, HDL-C, and LDL-C was 0.753 (95% CI, 0.679 to 0.827) for new-T2DM. When circulating miRNA-146a levels were added to the model, the AUC was increased to 0.844 (95% CI, 0.785 to 0.903, *P* = 0.0005 for the difference of AUCs with conventional model). After plasma HO-1 concentration and circulating miRNA-146a levels were introduced, the AUC increased significantly to 0.865 (95% CI, 0.813 to 0.917), compared with conventional model (*P* = 0.0001) or conventional model plus plasma HO-1 concentration (*P* = 0.0054).

We examined circulating miRNA-146a levels for possible association with concentrations of plasma HO-1 and HOMA-beta after natural log-transformation (Table 3). Higher circulating miRNA-146a levels were significantly associated with higher plasma HO-1 concentrations (β coefficient = 0.131, *P* < 0.001). The observed effect size of circulating miRNA-146a levels on plasma HO-1 concentrations did not change after adjusting for age, sex, and BMI (β coefficient = 0.135, *P* < 0.001). This association persisted after adjustment for other diabetes risk factors (β coefficient = 0.119, *P* < 0.001). We observed a significant inverse association between circulating miRNA-146a levels and HOMA-beta (multivariate β coefficient = -0.137, *P* = 0.023). However, no association was observed between circulating miRNA-146a and HOMA-IR.

**Table 3:** Associations of circulating miRNA-146 levels with plasma HO-1 concentrations and HOMA-beta.

MiRNA-146a levels*	HO-1 concentrations (ng/ml)*		HOMA-beta*	
	β (SE)	*P*	β (SE)	*P*
Crude	0.131 (0.030)	<0.001	-0.153 (0.062)	0.015
Adjusted, model 1†	0.135 (0.029)	<0.001	-0.152 (0.059)	0.011
Adjusted, model 2†	0.123 (0.029)	<0.001	-0.149 (0.060)	0.014
Adjusted, model 3†	0.119 (0.029)	<0.001	-0.137 (0.059)	0.023

* Natural log-transformed before analysis

† Model 1, adjusted for age, sex and BMI; Model 2, adjusted for Model 1, smoking, alcohol drinking, family history of diabetes and hypertension; Model 3, adjusted for Model 2, TC, TG, HDL-C and LDL-C

Abbreviations: BMI, body mass index; HDL-C, high-density lipoprotein-cholesterol; HO-1, heme oxygenase-1; HOMA-beta, homeostasis model assessment of beta cell function; LDL-C, low-density lipoprotein-cholesterol; TC, total cholesterol; TG, triglycerides.

## Discussion

In this study, we observed a significant positive association between levels of circulating miRNA-146a and risk of new-T2DM patients. When circulating miRNA-146a levels were added to the conventional model, the AUC was significantly increased. Additionally, circulating miRNA-146a levels were positively related to plasma concentrations of HO-1, a protein that may be targeted by miRNA-146a. Moreover, circulating miRNA-146a levels were inversely associated with HOMA-beta. However, no significant relationship between circulating miRNA-146a levels and HOMA-IR was detected. In our study, the relative levels of circulating miRNA-146a were calculated using the 2^-ΔΔCt^ method and the sample size was relatively small, therefore, the variations were quite wide.

MiRNA-146a, one of the inflammation-related miRNAs, contains fingerprints for various diseases and has been reported to predict immunological diseases and cancers, such as gastric cancer [31], hepatocellular carcinoma [32], and Sjogren’s syndrome [33]. In addition, miRNA-146a may also be involved in the pathological processes of inflammatory human degenerative diseases, such as Prion disease [34,35], Alzheimer’s disease [36,37] and Epilepsy [38,39].

However, few studies have investigated the relationship between this marker and diabetes pathogenesis. On the other hand, the role of miRNA-146a in the pathogenesis of inflammation and other degenerative aspects may make it participate in the course of T2DM. McMillan et al. reported that in RelB-depleted fibroblasts, after miR-146a overexpression, IL-1β-induced IL-6 production was also significantly reduced [40]. In addition, in human gingival fibroblasts, the inhibition of miRNA-146a resulted in increased IL-1β, IL-6 and TNF-α secretion, which suggested that miRNA-146a functions as a negative regulator of periodontal inflammation [41]. Moreover, miRNA-146a-mediated down-regulation of interleukin-1 receptor-associated kinase-1 together with NF-κB-induced up-regulation of interleukin-1 receptor-associated kinase-2 expression could promote an extensively sustained inflammatory response in hippocampus and neocortex of Alzheimer disease brain and in interleukin-1β and amyloid-β-42 peptide-stressed human astroglial cells [36]. The positive association between circulating miRNA-146a levels and new-T2DM risk observed in our study is consistent with that from a previous case-control study conducted in a Chinese sample [42]. However, our study had a much larger sample size and used persons participating in routine health examinations as controls, instead of T2DM-susceptible individuals with normal glucose tolerance. In addition, our study found that circulating miRNA-146a levels were inversely associated with HOMA-beta.

In a study conducted in Italy, Zampetaki et al. [19] identified no notable association between circulating miRNA-146a levels and T2DM, using microarray screening of pooled plasma samples of diabetes cases and controls. Several factors may explain the discrepancies between our findings and this study. First, our study was focused on miRNA-146a levels, whereas Zampetaki et al. conducted an exploratory analysis of multiple microRNAs. Second, differences in race and ethnicity may contribute to the divergence. It is well-known that race and ethnicity are reflected in distinct sequencing of the human genome and the mapping of human genetic variation [43]. As a key regulator of gene expression, the expression profiles of miRNAs probably vary among participants of different races or ethnicities. Finally, we considered only new-T2DM patients as cases to avoid possible confounding effects of anti-diabetic medications, while the diabetes cases in Zampetaki et al. were diagnosed during a certain period of follow-up time.

Heme oxygenase-1 (HO-1) has been found to exert its anti-inflammatory, anti-oxidant, and anti-proliferative effects on plenty of physiological and pathological processes [24]. Our previous study suggested that elevated plasma HO-1 concentrations were associated with higher risk for new-T2DM, likely in a dose–response manner [25]. Recently, Kozakowska and colleagues sought to examine the relationship between HO-1 and miRNA processing [26]. It was brought to light that activation of HO-1 in C2C12 cells increased production of miR-146a, regardless of the mode of stimulation. This view is corroborated by our findings that circulating miRNA-146a levels were correlated with plasma HO-1 concentrations and that the expression of miRNA-146a was notably higher in new-T2DM patients than controls. However, whether increased circulating miRNA-146a is caused by increased plasma HO-1 concentrations or other mechanisms remains unknown. On the other hand, Balasubramanyam and colleagues evaluated the role of miR-146a expression in peripheral blood mononuclear cells (PBMC) in relation to glycemic control and insulin resistance in both T2DM patients and non-diabetic subjects [44]. Intriguingly, the expression levels of miR-146a were significantly decreased in PBMCs from T2DM patients compared with cells from control subjects. The sources of circulating miRNAs may contribute to the above discrepancy. Consensus has not yet been reached, but several studies have addressed whether circulating miNRAs are derived not only from blood cells but also from tissues affected by disease [45].

Some limitations of this study should be acknowledged. First, we focused solely on the association between circulating miRNA-146a levels and new-T2DM and did not explore other miRNAs. In addition, the stratified analyses by some risk factors, such as family history of diabetes, were hampered due to the small numbers of participants available in subgroups. Furthermore, the expression levels of miRNA-146a may be affected by multiple factors. In our study, circulating miRNA-146a levels were found to be correlated with plasma HO-1 concentrations. However, we could not establish a cause-effect relationship or provide insight into the molecular mechanisms underlying the regulatory action of circulating miRNA-146a.

In conclusion, we found that circulating miRNA-146a levels were elevated in new-T2DM patients compared with controls. This association needs to be confirmed in future studies. In addition, the biological mechanisms and whether expression of circulating miRNA-146a holds predictive value for T2DM warrants further investigations.
